# State paid sick leave mandates associated with increased mental health disorder prescriptions among Medicaid enrollees

**DOI:** 10.1093/haschl/qxae045

**Published:** 2024-04-23

**Authors:** Johanna Catherine Maclean, Ezra Golberstein, Bradley Stein

**Affiliations:** Schar School of Policy and Government, George Mason University, Arlington, VA 22201, United States; National Bureau of Economic Research, Cambridge 02138, MA, United States; Institute of Labor Economics, 53113 Bonn, Germany; Division of Health Policy and Management, University of Minnesota School of Public Health, Minneapolis, MN 55455, United States; RAND, Pittsburgh PA, 15213, United States

**Keywords:** Medicaid, mental health, prescriptions, social insurance

## Abstract

The United States does not have a federal paid sick leave policy. As a result, many workers, in particular lower wage workers, cannot take time off work to attend to health and family responsibilities. Fifteen states have adopted or announced paid sick leave mandates that offer employees approximately 7 days of financially protected work time each year. This time can facilitate health care use, including treatment related to mental health disorders, conditions for which treatment is time-consuming. We studied the effect of state paid sick leave mandates on prescription medications dispensed for mental health disorders using the Medicaid State Drug Utilization Database 2011–2022. We found that medications dispensed for mental health disorders increased 6% per year following adoption of a state paid sick leave mandate.

## Introduction

Mental health disorders are common chronic conditions in the United States. In 2021, 22.8% of adults experienced any mental illness (57.8 million people).^1^ These disorders have profound and negative impacts on health and overall well-being. The costs of serious mental health disorders alone to the United States are estimated to be over $550 billion annually (in 2023 dollars), including costs associated with health care use, disability, crime, and a less productive workforce.^2^

Effective treatments are available for mental health disorders. Medicaid, the largest US purchaser of mental health disorder treatment,^3^ plays a central role in supporting access to effective treatment for the millions of Medicaid-enrolled Americans with these disorders. Prescription medications are critical components of evidence-based treatment for most major mental health disorders. Medicaid covers these treatments with, at most, very low patient cost-sharing.^4^

Unfortunately, many people with mental health disorders, including Medicaid enrollees, do not receive treatment for these conditions.^5^ For example, in 2021, fewer than 50% of people who could benefit from mental health care received any related treatment. There are many reasons for not receiving care, including costs and inability to pay, cultural and linguistic barriers, capacity constraints within the mental health disorder treatment system, stigma, and lack of readiness for treatment. A potentially important barrier for people who are employed, or dependents of the employed, is the inability to take time away from work (without losing wages) to receive treatment as many health care appointments are available during the standard workday. A review of over 2.5 million office visits shows that the most common times are during work hours: 10 Am and 2 Pm.^6^ In 2021, 19% of adults needing, but not receiving, mental health care indicated in a national survey that not having time to seek care was a barrier.^7^ Moreover, 30% reported that they did not know where to go for care and 5% reported transportation problems,^7^ barriers potentially mitigated by additional time to seek treatment. Employed Medicaid enrollees may also worry about stigma and the negative effects on their job if they request time away from work or reveal they are asking an employer for time off to receive care for a mental health disorder.

The United States is just 1 of 2 Organization for Economic Co-operation and Development countries without a federal paid sick leave (PSL) policy, despite 84% of Americans supporting PSL.^8^ In 2018 almost 40% of US employees reported that they did not have access to PSL through their employer.^9^ Thus, millions of working Americans must give up wages to take time away from work to receive health care for themselves or to support their dependents’ receipt of care—for example, by attending health care professional visits with a family member seeking treatment. Without government mandates, lower wage, part-time, and non-unionized workers in nonprofessional jobs are less likely than other workers to have access to PSL through their employer,^10^ suggesting that Medicaid enrollees who work may be particularly vulnerable to time-related barriers to health care use, for themselves or their dependent children. In 2021, 63% of nonelderly adult Medicaid enrollees worked for pay^11^ and 44% had children aged 18 years and younger in their home.^12^ Thus, PSL mandates could potentially impact working Medicaid enrollees themselves in terms of their use of PSL for health and health care needs, or through non–Medicaid-enrolled parents who take leave in relation to their Medicaid-enrolled children's health or health care needs. Further, most PSL mandates allow employees to use paid leave for additional dependents (eg, spouses and domestic partners and grandparents), which suggests that workers gaining access to PSL through state mandates could support Medicaid-enrolled adult dependents’ needs.

In the absence of federal policy, some states mandate that employers offer PSL. These laws require that employers provide approximately 7 days of PSL annually. As of October 2023, 15 states have adopted or announced a PSL mandate.^13^ Recent studies using research methods designed to elicit causal estimates of policy impacts (in particular, difference-in-differences [DID] methods) show that PSL mandates increase access to and use of PSL^10,14^ without leading employers to curtail the offering of other benefits such as health insurance or wages^10^ and have been associated with improved health,^10,14-17^ increased use of preventive care,^18,19^ and reduced hospitalizations,^20^ but there is a paucity of evidence regarding their effects on care for individuals with mental health disorders.

To address this gap in the literature, this study used the 2011–2022 Medicaid State Drug Utilization Database (SDUD), containing all dispensed prescriptions financed by Medicaid in retail and online pharmacies for which the Medicaid program receives rebates from the Medicaid Drug Rebate Program (MDRP), to examine the effect of state PSL mandates on medications dispensed for mental health disorders among Medicaid enrollees. Medicaid enrollees who work may be particularly responsive to state PSL mandates—which allow workers to take time off work for health care without foregoing wages—as they are less likely to have access to PSL without a mandate and they face low cost-sharing for health care, including medications for mental health disorders. Further, Medicaid enrollees have higher rates of these disorders but many do not receive care each year.^5^

## Data and methods

### Study data

We used 2011 through 2022 data on dispensed Medicaid-financed prescriptions for which the Medicaid program receives rebates from the MDRP, including both fee-for-service and managed-care programs, from the Centers for Medicare and Medicaid Service’s (CMS's) SDUD.^21^ The data include all prescriptions dispensed in retail and online pharmacies that are financed, fully or partially, by state Medicaid programs. States are required to report quarterly data to CMS for the SDUD in order to participate in the MDRP. Similar to others,^22^ we used data starting in 2011, as prior to March 2010, states were only required to report data for fee-for-service Medicaid programs to CMS, and some states were slow to begin reporting managed-care data in 2010.

In the SDUD, data points (drug-state-year-quarters) with less than 11 fills are suppressed. We followed previous research and assigned a value of 5 (the midpoint of all possible suppressed values: 0 to 10) to suppressed observations.^4,23,24^ However, as sensitivity analyses demonstrate, results are robust to imputing either 0 or 10 for suppressed data points (ie, the highest and lowest possible values). We aggregated the data to the state-year level to smooth out noise in the quarterly data. As described below, we excluded Washington, DC, from the analysis. We have 600 observations in our analysis dataset.

**Figure 1.**
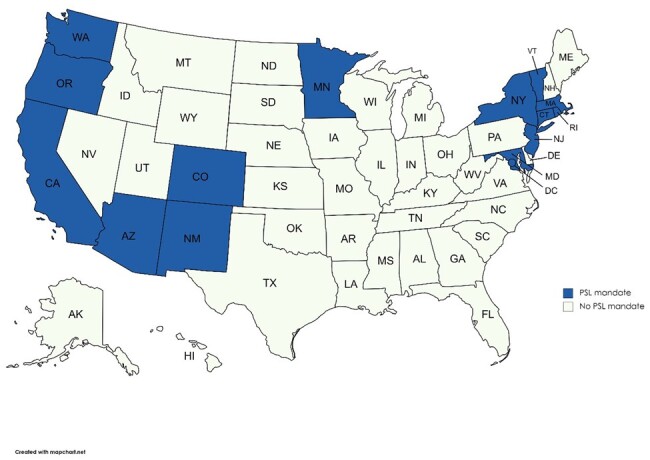
Distribution of state PSL mandates across US states, 2008–2023. Source: Authors’ analysis of data from the National Partnership for Women and Families, 2023. All state PSL mandates were effective or announced by October 2023, the most recent data available at the time of writing. The effective year in our study refers to the first full year in which a mandate is in place, so states that adopt a mandate after January 1st in a given year are coded as effective in the following year. Treatment states (effective years) are as follows: AZ (2018), CA (2016), CO (2021), CT (2012), DC (2009; note that DC is not included in our main analyses), MA (2016), MD (2019), MN (2024), NJ (2019), NM (2023), NY (2021), OR (2016), RI (2019), VT (2017), and WA (2018). Abbreviation: PSL, paid sick leave.

We used state PSL mandate data from the National Partnership for Women and Families,^13^ a nonpartisan research group that provides a database of all state PSL mandates in the United States. As of October 2023 (the most recent data available at the time of writing), 15 states (including DC) had adopted or announced a PSL mandate. Washington, DC, was the first state (we treat DC as state, but recognize this locality is a district) to adopt a PSL mandate in 2008. Minnesota is the most recent state to adopt a PSL mandate, which was effective January 1, 2024. All mandates require employers to cover time to receive care for employees and their immediate dependents (ie, children, spouses, partners). Most mandates allow paid sick time to be used for a broader set of dependents (eg, grandparents). There is limited monitoring of PSL use by employers, and employees generally are not required to provide employers with the reason for leave-taking. Figure 1 shows the geographic distribution of state PSL mandates to date; effective dates are listed in the Figure legend. We classified the first partial year in which the PSL mandate was adopted as the effective year.

Washington, DC, was excluded from the main analysis as this locality adopted a PSL mandate prior to 2011 and is therefore a “treated” control during our study period.^25^ Sensitivity analyses show that including DC does not lead to different findings.

We identified the effects of PSL mandates based on states that adopted mandates by 2022 in our DID regression. Thus, changes occurring after 2022 (ie, Minnesota) are not used in the DID analysis. (Although, as we describe later, we do use Michigan in event-study analyses.) State PSL mandates are relatively recent policies. Due to the newness of these policies, for several adopting states, we have a limited number of post-mandate years. Our findings are thus best interpreted as short-run effects based on the experiences of early PSL mandate-adopting states.

Four states did not have PSL mandates adopted or announced paid-time-off mandates that provide paid time off for all purposes by October 2023 (Illinois [2024], Maine [2021], Michigan [2019], and Nevada [2020]). Experts distinguish between paid-time-off and PSL mandates as the former confer fewer protections to employees. Paid-time-off mandates generally do not prohibit employer retaliation against employees who use (mandate-provided) PSL or have a requirement that the employee locate a replacement worker while on leave, do not allow employees to take time off without advance notice, and do not restrict the amount of documentation an employer can require the employee to provide to use PSL.^13^ For these reasons, we follow the National Partnership for Women and Families^13^ and treat PSL and paid-time-off mandates as distinct, but we control for paid-time-off mandates in all regressions.

### Outcome variables

We constructed annual counts (scaled by 1000) of dispensed prescriptions for major mental health disorders overall (ie, the sum of mental health disorder medications) and for mental health drug classes separately. We included medications in the following drug classes: attention-deficit/hyperactivity disorder medications, mood stabilizers, antidepressants, anti-anxiety medications, and antipsychotics. We chose not to convert counts to the rate per enrollee as there are differences across Medicaid populations in terms of access to mental health care professionals and likelihood of being diagnosed with a mental health disorder, which could be obscured by conversion to a rate. However, we show that results are not sensitive to conversion to a rate and we controlled for the number of enrollees per state-year in regressions. We interpret our findings as the change in the volume of dispensed medications financed by Medicaid overall, where the change may be driven by new patients or patients with prior treatment engagement (ie, extensive and intensive margins). The SDUD offers an overall count of dispensed medications only.

We modified the list of medications used in prior research,^4^ refined after review by a psychiatrist. We excluded medications indicated to treat mental health disorders if the majority of their use was likely for a non–mental health disorder (eg, clonidine for attention-deficit/hyperactivity disorder). Specific medications are listed in Table S1 (To access the appendix, click on the Details tab of the article online.)

We also examined the total costs of these dispensed prescriptions, and costs to state Medicaid programs and other payers (“patients”). We adjusted costs (scaled by $1 000 000) to 2022 dollars using the Consumer Price Index. The SDUD do not include rebates to state Medicaid programs from participating in the MDRP and therefore overstate costs to Medicaid. To account for this issue, we followed Maclean et al^23^ and adjusted payment estimates for average rebates assuming that state Medicaid programs recover 19% of Medicaid payments (the average rebate per state program estimated by Maclean et al).

### Statistical analyses

We investigated the effect of state PSL mandates using a DID framework. We compared trends in dispensed medication outcomes in states that do and do not adopt PSL mandates. We estimated an ordinary least squares (OLS) regression that controls for state PSL mandates (these mandates are lagged 1 year to allow for employees to accrue the benefit^13^ and to schedule and receive care where a prescription could be written), other paid leave mandates (family and medical leave and paid time off^26^), the effective minimum wage, the higher of the state or federal wage,^27^ recreational and medical marijuana laws,^28^ prescription drug-monitoring programs,^29^ Medicaid income-eligibility thresholds for parents and children ages 6 to 18 as a percentage of the Federal Poverty Level,^30^ Affordable Care Act (ACA) Medicaid expansion,^31^ monthly Temporary Aid to Needy Families (TANF) benefit ($) for a family of 4,^27^ political party of the governor,^27^ poverty rate,^27^ state demographics (age, sex, race, ethnicity, and educational attainment),^32^ number of mental health treatment provider offices and facilities,^33^ number of Medicaid enrollees,^27^ state fixed-effects, and year fixed-effects. We converted all monetary control variables to 2022 terms using the Consumer Price Index.

Data are weighted by the state Medicaid population^27^ and we clustered standard errors by the state.^34^ The University of Minnesota's Institutional Review Board determined this study to be Not Human Research.

### Sensitivity analyses

We conducted several sensitivity analyses to explore the robustness of our main findings. In sensitivity analyses, we used only the sum of dispensed mental health disorder medications as the outcome variable.

First, we estimated an “event-study,” which decomposes the main DID variable into a series of interaction terms between being a state that adopts or announces a PSL mandate by October 2023 and time-to-event, where the event is the PSL mandate effective date (the first partial year the mandate is in place). We assessed 5 years before policy adoption and 5 years after policy adoption in addition to the year of the event (PSL mandate adoption year). We coded states that adopt PSL mandates after 2022 (the last year of our study) in their pretreatment year (eg, since Minnesota adopted a PSL mandate in 2024, we coded this state as −2 in 2022, −3 in 2021, and −4 in 2020). We show that results are robust to treating states that adopt or announce a mandate after 2022 in the following 2 ways: (1) excluding these states and (2) coding these states as zero for all policy lead and lag variables—that is, as though they did not adopt a policy. In all event-study specifications, states that do not announce or adopt a PSL mandate by October 2023 are coded as zero for all event-time indicators.

Estimating an event-study offered us the opportunity to probe the key assumption of DID methods: that states adopting and not adopting a PSL mandate would have followed the same trends in outcomes post–PSL mandate if adopting states had not adopted the mandate. This assumption is untestable as we cannot observe counterfactual outcomes for PSL mandate–adopting states had they not adopted the mandate. However, examination of the coefficient estimates for years prior to policy adoption can offer suggestive evidence on the extent to which this assumption is likely to hold. If we observe similar adjusted trends for adopting and non-adopting states prior to the mandate (ie, coefficient estimates on the policy lead variables are small in size and not statistically different from zero), this pattern of results will support the validity of our design.

We also estimated state-specific trends in the outcome prior to the PSL mandate adoption in treatment states and over the full study period for other states; removed the estimated state-specific trend for each state from the value of the outcome; and re-estimated our main regression on the “de-trended” outcome. This approach allows us to further probe the importance of differential trends for adopting and non-adopting states.

To provide additional suggestive evidence on the validity of our DID design, we conducted a falsification test. We selected a health condition for which treatment is unlikely to be substantially impacted by financially protected work time made available through state PSL mandates. We selected dispensed medications for the treatment of brain cancer^35^; brain cancer is a serious health condition that can lead to death without treatment and thus we expected that medication treatment for this condition will be less impacted by PSL mandates than other conditions for which treatment is arguably more discretionary. We regressed the number of dispensed medications approved to treat brain cancer that appear in the SDUD (Table S1) on the PSL mandate and other controls.

We also implemented a range of robustness checks to assess whether results are driven by the specification and sample selected. Specifically, we (1) excluded time-varying covariates; (2) included US Census division-by-year fixed-effects rather than year fixed-effects; (3) controlled for the state unemployment rate among nonelderly adults^32^ and mental health outcomes among adults aged 18 years and older^36^; (4) did not lag the PSL mandate 1 year; (5) controlled for a bordering a state with a PSL mandate; (6) dropped first Maryland and Virginia, and then Connecticut, New Jersey, and New York given substantial numbers of people working and residing in different states in these 2 areas; (7) replaced the PSL indicator with an indicator taking on a value of 1 if a state has a PSL mandate or a paid-time-off mandate; (8) estimated unweighted regression; (9) dropped 2020 (the first pandemic year; in this year there were large-scale employment losses, which may impact access to PSL through state mandates and there was a federal PSL mandate in place^37^); (10) dropped states that adopted a PSL after 2019 as we have few post-treatment years for these states; (11) dropped years prior to 2014 (ie, pre-ACA Medicaid expansion) and dropped state/year pairs 2014–2022 in which ACA Medicaid expansion was not in place to generate a potentially more suitable comparison group (this exclusion led us to drop over half the sample); (12) included DC; (13) used an estimator proposed by Gardner^38^ that is robust to potential bias from dynamic and heterogeneous treatment effects when using DID regressions with a staggered policy roll-out (we excluded Connecticut from this analysis as we had a single pretreatment year for that state [which adopted a PSL mandate in 2012] and as we lagged the PSL mandate 1 year and thus have no pretreatment data to estimate a fixed-effects parameter for this state) and conducted a diagnostic test to assess the importance of these biases^25^; and (14) imputed suppressed data points in the SDUD with 0 (smallest possible value) or 10 (largest possible value).

Third, we estimate a “leave-one-out” analysis allowing us to test if results are driven by the experience of any specific state(s) that adopts a PSL mandate. More specifically, we sequentially dropped each state adopting a PSL mandate by quarter 4 of 2022 and re-estimated our regression. We also estimated a regression in which we use only California as the treated state as California has a number of sub-state PSL mandates.^13^

### Limitations

Our analysis is subject to several limitations, as follows:

1. The SDUD does not have any sub-state geographic information and thus we cannot isolate cities and counties with PSL mandates.^13^
2. We are unable to study use of medications or the characteristics of patients who may increase their medications post-mandate as we only observed dispensed prescriptions.
3. Our payment variables do not include information on rebates to Medicaid states from the MDRP and thus overstate what state programs paid for the medications we studied. However, we used estimates from Maclean et al^23^ to “scale” the estimates by the average rebate.
4. We only measured medications dispensed from retail and online pharmacies.
5. We do not have information on enrollee employment status in the SDUD and employment is important as PSL mandates (directly) impact the employed, and during COVID-19 many people lost their job, suggesting that this issue was particularly severe in 2020. However, in robustness checking, we controlled for state-level unemployment rates and we excluded the COVID-19 year (2020).
6. While PSL mandates could impact treatment use among Medicaid enrollees for the above-noted reasons, such effects are not guaranteed given the barriers that enrollees face in accessing care. For example, and in addition to barriers cited earlier, many providers do not accept Medicaid.^39,40^ Not all enrollees work,^11^ and among those who work, some may work in jobs that are exempt from PSL mandates. Hence, the extent to which PSL mandates benefit Medicaid enrollees is a priori unclear.

## Results

### Main results

Summary statistics for the overall sample and states that did and did not adopt or announce a PSL mandate by October 2023 are reported in Table 1. The number of mental health disorder dispensed prescriptions (per 1000) is 2866. Antidepressants (1201) and anti-anxiety (725) medications are the most commonly dispensed medications. Overall, dispensed prescription counts are somewhat higher in states that adopted or announced a PSL mandate, but there are differences across the 2 groups for specific drug classes. In particular, counts of dispensed attention-deficit/hyperactivity disorder prescriptions are higher in non-adopting states. Figure S1 reports unadjusted trends in dispensed prescriptions for mental health disorders for adopting and non-adopting states.

**Table 1.** Dispensed prescriptions for mental health disorder treatment per state, state policies, and state characteristics: 2011–2022.

Sample	All states	States that adopt a PSL, pre-policy	States that do not adopt a PSL
Prescription fills			
Mental health drugs	2867.7	3263.3	2103.5
Attention-deficit/hyperactivity disorder	219.7	152.0	275.6
Mood stabilizer	197.3	247.5	133.6
Antidepressant	1201.0	1315.1	819.4
Anti-anxiety	725.4	904.4	554.6
Antipsychotics	524.2	644.3	320.2
Treatment variable			
Paid sick leave mandate (lagged 1 year)	0.19	0	0
Control variables			
Paid time off mandate	0.016	0	0.028
Paid family and medical leave mandate	0.24	0.38	0
Effective minimum wage ($)	12.1	13.1	10.8
Recreational marijuana law	0.24	0.11	0.072
Medical marijuana law	0.62	0.86	0.38
Prescription drug-monitoring program	0.99	1	0.99
Medicaid income thresholds for children aged 6–18 (% Federal Poverty Level/100)	1.75	1.69	1.58
Medicaid income thresholds for parents (% Federal Poverty Level/100)	1.05	1.33	0.83
ACA Medicaid expansion	0.56	0.60	0.38
Medicaid beneficiaries	3 918 575	4 431 451	2 224 325
TANF 4-person family ($/month)	811.0	1144.7	585.4
Democrat governor	0.51	0.84	0.28
Poverty rate	12.9	13.3	13.5
Age 0–15 years	0.20	0.20	0.20
Age 16–44 years	0.38	0.39	0.38
Age 45 years and older	0.41	0.41	0.42
Female	0.51	0.51	0.51
Male	0.49	0.49	0.49
White	0.77	0.76	0.77
Black	0.13	0.11	0.15
Other race	0.11	0.13	0.072
Hispanic	0.19	0.23	0.13
Less than high school	0.15	0.16	0.15
High school	0.28	0.25	0.30
Some college	0.26	0.26	0.27
College degree or higher	0.30	0.32	0.28
Offices of mental health physicians	607.1	674.6	387.2
Offices of mental health non-physicians	1290.0	1247.6	843.2
Outpatient MH/SUD facilities	481.4	500.0	337.2
Residential MH/SUD facilities	386.1	510.9	194.1
MH/SUD hospitals	26.3	28.5	23.6
No.	600	97	432

Abbreviations: ACA, Affordable Care Act; MH/SUD, mental health/substance use disorder; PSL, paid sick leave mandate; TANF, Temporary Aid to Needy Families.

Source: Authors’ analysis of data from the State Drug Utilization Database, 2011–2022. The unit of observations is a state in 1 year. Washington, DC, is excluded from the sample. Data are weighted by the number of Medicaid enrollees in the state.

Our main results for the effect of state PSL mandates on Medicaid-financed dispensed prescriptions are reported in Table 2. Following adoption of a state PSL mandate, the number of mental health disorder dispensed prescriptions increased by 195 or 6% relative to the pre-policy mean in adopting states. In results not reported, we found that ACA Medicaid expansion led to an increase of 407 (per 1000) or 12% in dispensed mental health disorder medications, which is more than 2 times larger than our finding for PSL mandates, suggesting that our PSL mandate effect sizes are not implausible. Henceforth, we report relative (ie, %) changes in the text for brevity, but exact changes in outcomes are provided in all tables reporting regression results.

**Table 2.** Adjusted estimates of Medicaid-financed dispensed mental health disorder prescriptions and payments, 2011–2022.

Outcome variables	Coefficient estimate [95% CI] (*P* value)
Dispensed mental health disorder prescriptions (1000’s)	194.75*** [77.94, 311.57] (0.00)
Percentage change	5.97
Pretreatment mean in PSL mandate–adopting states	3263.26
Total payments for dispensed mental health disorder prescriptions ($1 000 000’s)	74.80^***^ [23.03, 126.57] (0.01)
Percentage change	15.48
Pretreatment mean in PSL mandate–adopting states	483.15
Medicaid payments for dispensed mental health disorder prescriptions ($1 000 000’s)	74.91^***^ [22.47, 127.34] (0.01)
Percentage change	15.84
Pretreatment mean in PSL mandate–adopting states	472.85
Patient payments for dispensed mental health disorder prescriptions ($1 000 000’s)	−0.11 [−2.00, 1.79] (0.91)
Percentage change	−1.07
Pretreatment mean in PSL mandate–adopting states	10.29
No.	600

Abbreviation: PSL, paid sick leave.

Source: Authors’ analysis of data from the State Drug Utilization Database, 2011–2022. Outcome variables are the number of dispensed medications and payments. Dispensed medications are scaled by 1000 and payments are scaled by 1 000 000. The unit of observations is a state in 1 year. Washington, DC, is excluded from the sample. All regressions estimated with ordinary least squares (OLS) and control for time-varying state characteristics, state fixed-effects, and year fixed-effects. Data are weighted by the number of Medicaid enrollees in the state. 95% CIs (*P* values) that account for within-state clustering are reported in square brackets (parentheses). ***Statistically different from zero at the 1% level.

In Table 2, we also report the impact of state PSL mandates on total, Medicaid, and patient payments for dispensed prescriptions for mental health disorders. Post–PSL mandate, total payments adjusted for MDRP rebates^23^ increased by 15% and state Medicaid program payments increased by 16%. Patient payments (which reflect just 2% of all payments) did not change following adoption of a state PSL mandate.

In Table 3, we separately consider dispensed prescriptions for medications associated with specific mental health disorders. We reproduce our overall medication counts for comparison. Our overall findings for medications for mental health disorders are driven by changes in attention-deficit/hyperactivity disorder medications (24%), mood stabilizers (3%), anti-anxiety medications (7%), and antipsychotics (10%). There was no statistically significant change in antidepressant dispensed medications post-mandate, although the coefficient estimate carries a positive sign. We also report *P* values that are corrected for multiple comparisons following a procedure developed by Anderson.^41^

**Table 3.** Adjusted estimates of Medicaid-financed dispensed mental health disorder prescriptions by specific drug classes (1000’s), 2011–2022.

Drug class	Coefficient estimate [95% CI] (*P* value)
Dispensed mental health disorder prescriptions (1000’s)	194.75^***^ [77.94, 311.57] (0.00) {0.01}
Percentage change	5.97
Pretreatment mean in PSL mandate–adopting states	3263.26
Dispensed attention-deficit/hyperactivity disorder prescriptions	37.20^**^ [4.33, 70.07] (0.03) {0.04}
Percentage change	24.48
Pretreatment mean in PSL mandate–adopting states	151.98
Dispensed mood stabilizer prescriptions	6.82^**^ [0.47, 13.18] (0.04) {0.04}
Percentage change	2.76
Pretreatment mean in PSL mandate–adopting states	247.54
Dispensed antidepressant prescriptions	29.35 [−30.96, 89.67] (0.33) {0.13}
Percentage change	2.23
Pretreatment mean in PSL mandate–adopting states	1315.06
Dispensed anti-anxiety prescriptions	59.06^*^ [−11.16, 129.29] (0.10) {0.06}
Percentage change	6.53
Pretreatment mean in PSL mandate–adopting states	904.35
Dispensed antipsychotic prescriptions	62.31^***^ [33.20, 91.42] (0.00) {0.00}
Percentage change	9.67
Pretreatment mean in PSL mandate–adopting states	644.34
No.	600

Abbreviation: PSL, paid sick leave.

Source: Authors’ analysis of data from the State Drug Utilization Database, 2011–2022. Outcome variables are the number of dispensed prescriptions scaled by 1000. The unit of observations is a state in 1 year. Washington, DC, is excluded from the sample. All regressions estimated with ordinary least squares (OLS) and control for time-varying state characteristics, state fixed-effects, and year fixed-effects. Data are weighted by the number of Medicaid enrollees in the state. 95% CIs (*P* values) that account for within-state clustering reported in square brackets (parentheses). *P* values corrected for multiple comparisons are reported in braces. ^***, **, *^Statistically different from zero at the 1%, 5%, and 10% level, respectively.

### Sensitivity results

We report results from event-study analyses, using 3 alternative approaches to handling states that adopted a PSL mandate after the end of our study period (2022), which do not provide evidence of differential trends in mental health disorder medications in states that adopted and did not adopt a PSL mandate. This finding supports our use of DID as a credible research design to estimate the effects of PSL mandates on Medicaid-financed dispensed prescriptions for mental health disorders. We further show that our results are robust to using the de-trended count of mental health dispensed medications.

We conducted a falsification test, where we examine a medication obtained in a retail setting for which PSL mandates should be expected to have no impact. We considered the impact of state PSL mandates on medications that, without use, life expectancy is extremely short—namely dispensed medications for brain cancer. We found no statistically significant evidence that dispensed medications for this health condition change following a PSL mandate adoption.

Our results are broadly robust to different samples and specifications. We note that excluding California from the sample reduces the PSL mandate coefficient estimate substantially. California is the largest state in the nation in terms of overall population, Medicaid enrollees, and economy^27^; thus, we would expect this state to be empirically important. Further, California's PSL mandate is relatively generous in terms of included workers and dependents,^13^ which further suggests that this state would be important empirically. A decomposition of our overall DID regression coefficient, which is composed of all possible 2-by-2 comparisons of states that changed and did not change their PSL mandate status^25^ (results not shown), shows that 92% of all comparisons are “reasonable” in that we do not use previously treated states as a comparison for later treated states, which suggests our setting is not one where we would expect such biases to be concerning. Finally, our results do not appear to be driven by the experiences of 1 or 2 states.

## Discussion

Access to health care depends on a variety of factors.^42^ As such, approaches to improve on enduring rates of underuse of mental health disorder treatment need to address a range of barriers. In this study, we examined the effects of state PSL mandates on dispensed prescription medications for mental health disorders in state Medicaid programs. These mandates can reduce time-related barriers to treatment and could be part of a broader suite of policies adopted to address underuse of mental health disorder treatment among enrollees. Medicaid is the biggest payer of mental health disorder treatment nationally,^3^ and people with these health disorders are disproportionately likely to be enrolled in Medicaid.^43^

Medicaid is a notable setting to investigate barriers to mental health disorder care, since cost-sharing, normally a key barrier,^7^ is minimal to nonexistent for enrollees. Even though enrollees have limited direct financial costs of treatment, they still may face substantial barriers from the time and effort costs associated with accessing treatment. For example, recent research finds that the expansion of public transportation led to reduced no-show health care appointments, with the greatest effects for Medicaid enrollees.^44^

We found that after states implemented a PSL mandate, the number of prescriptions dispensed for mental health disorder drugs increased by 6% relative to pre–PSL mandate levels. These findings capture the effect of PSL mandated by states independent of the myriad factors that may dissuade enrollees from accessing this care. We also found that the adoption of PSL mandates is associated with relative increases in Medicaid spending. These cost increases to Medicaid should be considered alongside the considerable expense of untreated, or undertreated, mental health disorders. Better management of these conditions through the use of psychotropics may lead to downstream cost-savings if more intensive and expensive care is averted, or by addressing unmet treatment need, which can lead to substantial social costs (eg, general health care, crime, social service use). Moreover, improvements in the overall quality of life for enrollees who are able to receive care is also a benefit. For example, increasing the primary care reimbursement rate in Medicaid has been shown to cost-effectively improve mental health disorder outcomes among enrollees.^45^ Paid sick leave, by facilitating treatment, may lead to similar cost offsets.

We note the possibility that PSL mandates could lead some patients to substitute counseling for medications as counseling is more time-intensive, but the reverse pattern of substitution is less likely. Such patterns, if occurring, could impact the interpretation of our findings. Medicaid enrollees with particularly severe mental health disorders are unlikely to be directly impacted by PSL mandates as they are not likely to work, although their family members and caregivers (who may work) could benefit from such policies as state PSL mandates, to date, allow workers to use the benefits to care for family members .^26^ Other policies will likely be needed to address unmet need for care among this population.

This study relies on aggregated data on prescription drugs dispensed. Future research could assess the effects of PSL mandates on broader patterns of mental health disorder treatment using individual-level data. This study uses early PSL-adopting states for identification of treatment effects, and future studies could revisit this question, after more states have adopted mandates, to re-assess this relationship. Another avenue for future research would be to study which patients are experiencing changes in mental health care use—for example, do PSL mandates allow new patients to receive care (extensive margin effects) or do these policies facilitate use of additional care by established patients (intensive margin effects)? Similarly, separating changes in use by employees and their dependents would further our understanding of how, and for whom, PSL mandates benefit in terms of mental health care use would be an interesting research objective. Finally, examining which attributes of PSL mandates are most important for health and health care outcomes would be a useful area of study.

## Conclusion

We found that state-level PSL mandates adopted between 2011 and 2022 increased dispensed prescriptions for mental disorders among Medicaid enrollees. These findings indicate that providing financially protected work time to receive health care can allow people to receive valuable care necessary to treat chronic conditions that impose substantial costs.

## Supplementary Material

qxae045_Supplementary_Data
