# Supplementary material for: State paid sick leave mandates associated with increased mental health disorder prescriptions among Medicaid enrollees
Source: Health Aff Sch. 2024 Apr 23;2(5):qxae045. doi: 10.1093/haschl/qxae045 (PMC11068101; doi:10.1093/haschl/qxae045)
Supplement: qxae045_Supplementary_Data [file qxae045_supplementary_data.zip › Supplementary Table 1.docx]

Supplementary Table 1. Medications included in the analysis, 2011-2022.

| Drug class | Drug name |
| --- | --- |
| Attention-deficit hyperactivity disorder | adderall, aptensio, concerta, cotempla, daytrana, dexedrine, intuniv, kapvay, metadate, methylin, procentra, quillichew, quillivant, ritalin, Strattera, vyvanse |
| Mood stabilizer | depakote, divalproex, equetro, eskalith, lamictal, lithium, lithobid, valproate |
| Anti-depressant | amoxapine, aplenzin, brintellix, budeprion, bupropion, celexa, citalopram, cymbalta, dermacinrx, drizalma, duloxetine, effexor, elavil, fetzima, fluoxetine, forfivo,  imipramine, khedezla, lexapro, marplan, nardil, nefazodone,  norpramin, pamelor, parnate, paroxetine, paxil, pexeva, phenelzine, pristiq, prozac, remeron, sertraline, surmontil, tofranil, trintellix, viibryd, vivactil, wellbutrin, zoloft, zulresso |
| Anti-anxiety | alprazolam, ativan, buspar, buspirone, clonazepam, diastat,  diazepam, klonopin, librium, lorazepam, oxazepam, tranxene, valium, xanax |
| Anti-psychotics | abilify, clozapine, clozaril, fazaclo, geodon, haldol, invega, latuda, olanzapine, prolixin, quetiapine, risperdal, seroquel, thorazine, trilafon, zyprexa |
| Brain cancer | afinitor, alymsys, avastin, bicnu, carmustine, danyelza, everolimus, gliadel, lomustine, mekinist, temodar, zirabev |

Source: Authors’ analysis of data from the State Drug Utilization Database, 2011-2022.

Notes: See text for data source.
