# Supplementary material for: State paid sick leave mandates associated with increased mental health disorder prescriptions among Medicaid enrollees
Source: Health Aff Sch. 2024 Apr 23;2(5):qxae045. doi: 10.1093/haschl/qxae045 (PMC11068101; doi:10.1093/haschl/qxae045)
Supplement: qxae045_Supplementary_Data [file qxae045_supplementary_data.zip › Supplementary Table 2.docx]

Supplementary Table 2. Adjusted estimates of Medicaid-financed dispensed mental health disorder prescriptions de-trending the data, using a falsification outcome, taking the rate of dispensed prescriptions per 100,000 Medicaid enrollees, and using a Poisson regression, 2011-2022.

| Outcome variable: | Coefficient estimate  [95% confidence interval] |
| --- | --- |
| Dispensed mental health disorder | 189.71*** |
| prescriptions (1,000s de-trended outcome)┼ | [63.29,316.13] |
|  | (0.00) |
| Percent change | 5.84 |
| Pre-treatment mean in PSL mandate adopting states | 3246.76 |
| N | 588 |
| Dispensed brain tumor prescriptions | -869.17 |
| (1,000s falsification outcome) | [-3,954.66,2,216.33] |
|  | (0.57) |
| Percent change | -10.03 |
| Pre-treatment mean in PSL mandate adopting states | 8662.43 |
| N | 600 |
| Dispensed mental health disorder | 6,359.48** |
| prescriptions (rate per 100,000) | [588.47,12,130.49] |
|  | (0.03) |
| Percent change | 7.06 |
| Pre-treatment mean in PSL mandate adopting states | 90061.19 |
| N | 600 |
| Dispensed mental health disorder | 202895.96*** |
| prescriptions (count using a Poisson)┼┼ | [85,138.42,320653.50] |
|  | (0.00) |
| Percent change | 6.22 |
| Pre-treatment mean in PSL mandate adopting states | 3263264.54 |
| N | 600 |

Source: Authors’ analysis of data from the State Drug Utilization Database, 2011-2022.

Notes: Outcome variables are the number of dispensed medications. The unit of observations is a state in a year. DC is excluded from the sample. All regressions estimated with OLS and control for time-varying state characteristics, state fixed effects, and year fixed effects unless otherwise noted. Data are weighted by the number of Medicaid enrollees in the state. 95% confidence intervals (p-values) that account for within-state clustering reported in square brackets (parentheses).

┼Connecticut, which adopted a PSL mandate in 2011, is excluded from the sample as there is insufficient data to de-trend data for that state.

┼┼The number of Medicaid enrollees is the exposure variable.

***,**, and * = statistically different from zero at the 1%, 5%, and 10% level.
