# Supplementary figures and images for: State paid sick leave mandates associated with increased mental health disorder prescriptions among Medicaid enrollees
Source: Health Aff Sch. 2024 Apr 23;2(5):qxae045. doi: 10.1093/haschl/qxae045 (PMC11068101; doi:10.1093/haschl/qxae045)

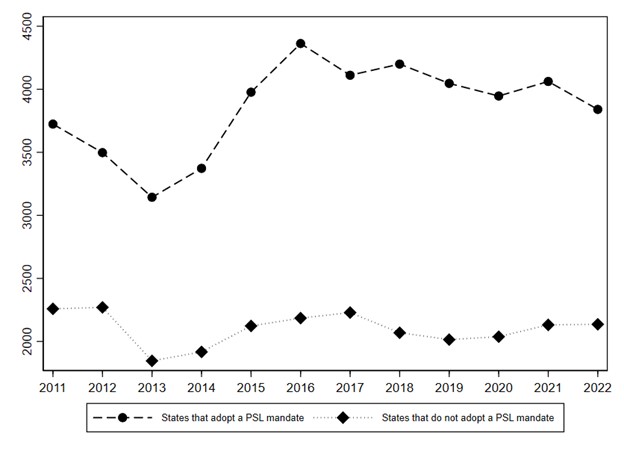

Supplement: qxae045_Supplementary_Data [file qxae045_supplementary_data.zip › supplementary_figure1.jpg]

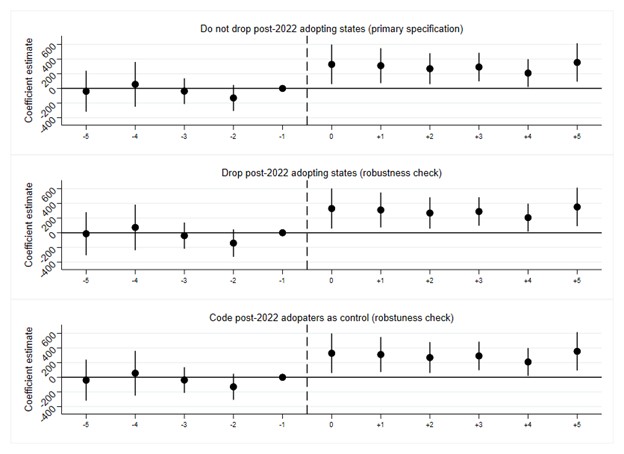

Supplement: qxae045_Supplementary_Data [file qxae045_supplementary_data.zip › supplementary_figure2.jpg]

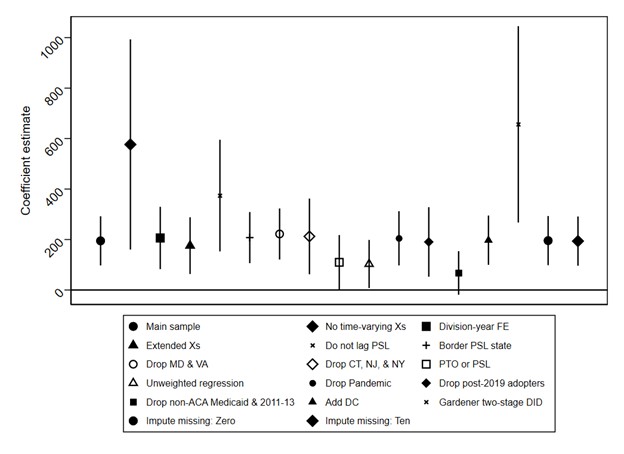

Supplement: qxae045_Supplementary_Data [file qxae045_supplementary_data.zip › supplementary_figure3.jpg]

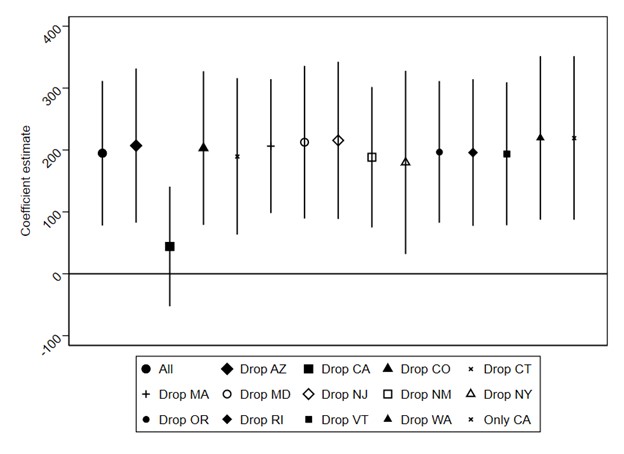

Supplement: qxae045_Supplementary_Data [file qxae045_supplementary_data.zip › supplementary_figure4.jpg]
